# Prevalence of apical periodontitis and endodontic treatment in a Kosovar adult population

**DOI:** 10.1186/1472-6831-11-32

**Published:** 2011-11-29

**Authors:** Blerim Kamberi, Veton Hoxha, Miranda Stavileci, Edmond Dragusha, Astrit Kuçi, Lumnije Kqiku

**Affiliations:** 1 Department of Dental Pathology and Endodontics, University Dentistry Clinical Center of Kosovo, Prishtina, Kosovo; 2 Division of Preventive and Operative Dentistry, Endodontics, Pedodontics and Minimally Invasive Dentistry, Department of Dentistry and Maxillofacial Surgery, Graz, Austria

## Abstract

**Background:**

Despite numerous studies on the prevalence of apical periodontitis (AP) and endodontic treatment in diverse geographical populations, there are currently no data on the prevalence of these conditions in populations of adults native to Kosovo. Therefore, little is known about how widespread these conditions are, and whether there is any correlation between root canal treatment and AP. The purpose of our research was to address this anomaly by investigating AP and endodontic treatment in an adult Kosovar population based on radiographic examination.

**Methods:**

The sample used for this study consisted of randomly selected individuals referred to the University Dentistry Clinical Center of Kosovo in the years 2006-2007. Orthopantomographs of 193 patients were evaluated. The periapical status of all teeth (with the exception of third molars) was examined according to Ørstavik's Periapical Index. The quality of the root canal filling was rated as 'adequate' or 'inadequate' based on whether all canals were filled, the depth of fill relative to the radiographic apex and the quality of compaction (absence/presence of voids). Data were analyzed statistically using the Chi-square test and calculation of odds ratios.

**Results:**

Out of 4131 examined teeth, the prevalence of apical periodontitis (AP) and endodontic treatment was 12.3% and 2.3%, respectively. Of 95 endodontically-treated teeth, 46.3% were associated with AP. The prevalence of AP increased with age. The prevalence in subjects aged over 60 years old (20.2%) was higher than in other age groups. A statistically significant difference was found for the frequency of endodontically-treated teeth associated with AP in the 40-49 year age group (P < 0.001). Of some concern was the discovery that only 30.5% of the endodontically-treated teeth examined met the criteria of an acceptable root canal filling. Inadequately root-filled teeth were associated with an increased AP risk.

**Conclusions:**

The prevalence of AP and the frequency of endodontically-treated teeth with AP in this Kosovar population are higher than those found in other countries. Inadequate root canal fillings were associated with an increased prevalence of AP.

## Background

Apical periodontitis (AP) is a multifactorial condition resulting from the interaction of many factors, predominantly bacteria [1]. It is characterized by a reaction of the periapical tissues to irritants diffusing at relatively low intensity and over an extended duration from an inflamed or necrotic pulp or a failed endodontic treatment [2]. Bacteria and their toxins can reach the pulp space *via *dental caries, trauma, or operative procedures [3,4] and can then advance into the periapical tissues, where they meet the various factors of the host defense systems [5].

The biological and therapeutic aim of endodontic treatment is either to prevent AP or to create optimal conditions for healing, based on the removal of infection and elimination of bacteria from the root canal system and prevention of re-infection. Endodontic treatment is widely recognized as a highly intricate task and epidemiological studies report that the frequency of teeth containing poor quality endodontic treatments is high [4-6]. Failure of a root filling is associated with inadequate endodontic treatment, through either technical error or insurmountable difficulty in the canal system of the tooth in question [7,8]. In several epidemiological studies, poor quality endodontic treatment was found to be associated with AP [9-11], which is commonly observed in root-filled teeth. The healing rate of patients experiencing AP after endodontic treatment in a general practice has been estimated to be as low as 50-75% [6,9,12,13].

Many authors have used clinical and radiologic criteria in assessing the quality of endodontic treatment and its correlation with apical lesions [14-16]. Apical radiography provides important information on the potential progression, regression and/or persistence of AP [17,18].

The literature contains a number of studies that present data regarding the prevalence of AP and endodontically-treated teeth, and these vary with regard to study populations, radiographic methods and classifications of AP used [9-11,19-26].

However, despite information from other ethnic groups, studies on periapical health have not yet been performed in Kosovo. No data are available on the prevalence of AP in either unfilled or root-filled teeth in the Kosovar population. The purpose of this study was therefore to use radiographic examination to investigate the prevalence of AP and endodontic treatment in an adult Kosovar population and to reveal any correlation between these conditions.

## Methods

### Sample Selection

The protocols used in this study have been approved by the Ethical Board of the University Dentistry Clinical Center of Kosovo, in Prishtina, Kosovo.

The sample used for this study consisted of randomly selected individuals referred by general dentists to the University Dentistry Clinical Center of Kosovo in the years 2006-2007. Orthopantomographs (OPGs) were selected by an independent observer. Radiographs of patients less than 18 years of age and/or with less than 10 standing teeth (including third molars) were excluded. Also excluded were any OPGs that were damaged in any way or were of poor quality (i.e. poor coverage of the periapical region, marked changes in radiographic density and images suggestive of unrelated periodontal/endodontic disease and/or post-endodontic surgical lesions). After applying the exclusion criteria, 193 OPGs were included in the study. These OPGs were examined by two independent observers. Patient-related information was limited only to age and gender, thus maintaining patient confidentiality. Following sample collection, radiographs were distributed by gender and age group. The OPGs used in this study were taken by a trained radiology assistant using an X-ray generator (PM 2002 CC Proline, Planmeca, Helsinki, Finland) and Kodak dental films (T - MATE, Kodak, New York, USA). All films were processed in a XR 24 Novamachine (Durr Dental, Bietigheim, Germany) using Durr Dental developer and fixer.

### Radiographic examination

All OPGs were assessed in optimal viewing conditions where the surrounding (environmental) lighting was controllable to achieve the maximum radiographic contrast. OPGs were placed on a viewing screen and the area surrounding the OPG was blocked with a dark material to block the lateral light and improve viewing contrast. To enhance the image, magnification (3×) was used on all radiographs. Prior to evaluating the OPGs, both examiners underwent a course of calibration for the Periapical Index (PAI) [17] using 100 retroalveolar radiographs, as recommended by the creators of this index.

### Assessment of apical periodontitis

Teeth were categorized as endodontically treated if they had been obturated with a radio-opaque material in the pulp chamber and/or in one or more of the root canals. Radiographs were assessed for the presence and severity of AP using the method of Orstavik et al (1986) [17]. Briefly, apical periodontitis was judged present in teeth in which the apical part of the periodontal space was less than twice the remaining lateral ligamental space and in which a radiolucency of more than twice the width of the lateral periodontal ligament space was associated with the apical portion of the root. Apical status was assessed using the PAI score [17], according to which 5 scores were attributed to the apical area of the radiographic images, as follows: 1) normal periapical structures; 2) small changes in bone structure; 3) changes in the bone structure with little mineral loss; 4) periodontitis with well-defined radiolucent area; 5) severe periodontitis with exacerbating features.

For multirooted teeth, the root with the highest PAI score was recorded. For teeth scored 3, 4 and 5, i.e., those with chronic apical periodontitis, the abbreviation "AP" was used. For teeth with AP associated with endodontic treatment, the abbreviation "AP/ET" was used.

### Assessment of endodontic treatment of teeth

The criteria used for evaluation of the quality of the root filling were modified slightly from those described by Tronstad *et al*. [27] and Tavares et al. [28], as follows: 1. Adequate: all canals obturated, no voids present, root canal fillings terminate 0-2 mm short of the radiographic apex. 2. Inadequate: root canal fillings end > 2 mm short of the radiographic apex or are grossly overfilled (i.e. extrusion of filling material through apex), root canal fillings with voids, inadequate density, unfilled canals, and/or poor compaction.

### Statistical analysis

Statistical evaluation of the data was performed using the statistical package, InStat 3 (GraphPad, San Diego, USA; demonstration version, publicly available to download at http://www.graphpad.com/demos/). The Chi-square test was used to determine the significance of differences by sex, age, dental arch (maxillary/mandibular) and region for the following parameters: number of teeth with AP; number of endodontically treated teeth; and the number of endodontically treated teeth with AP. P < 0.05 was accepted as statistically significant.

## Results

The inter-examiner agreement was determined by the Cohen's Kappa for the scores of all teeth (kappa = 0.88).

The average patient age was 34.5 ± 11.2 years. The distribution of female and male subjects is given in Table 1, sub-divided by age group. There was no significant difference between males and Females in the total number of teeth present.

**Table 1:** Distribution of patients by age group and sex

Age group	Male	Female	Total (%)
< 20	8	16	24 (12.4%)
20-29	18	30	48 (24.9%)
30-39	28	38	66 (34.2%)
40-49	13	14	27 (14.0%)
50-59	6	11	17 (8.8%)
≥60	7	4	11 (5.7%)

**Total (%)**	**80 (41.5%)**	**113 (58.5%)**	**193 (100.0%)**

The overall prevalence of AP in the examined teeth was 12.3% (Table 2). Of 4131 examined teeth, 95 were endodontically treated (2.3%). The prevalence of endodontic treatment was significantly higher in males (3.0%), than in females (1.8%) (P < 0.05). There were no statistically significant differences between males and females for the number of teeth with AP or ET/AP (P > 0.05).

**Table 2:** Distribution of total number of teeth, teeth with AP, ET, AP/ET by sex

Gender	No of teeth	Teeth with AP (%)	Teeth with ET (%)*	Teeth w. AP/ET (%)
Male	1718	225 (13.1%)	52 (3.0%)	20 (38.5%)
Female	2413	284 (11.8%)	43 (1.8%)	24 (55.8%)

**Total**	**4131**	**509 (12.3%)**	**95 (2.3%)**	**44 (46.3%)**

*Statistically significant difference between males and females: P < 0.05

AP, apical periodontitis; ET, endodontically treated teeth; AP/ET, apical periodontitis in endodontically treated teeth

The prevalence of AP increased with age (Table 3), with the highest prevalence in the ≥60 years group. There was a statistically significant difference among the age groups in the frequency of endodontically treated teeth with AP (P < 0.01). The 20-29 and 40-49 years age groups had a significantly higher prevalence of endodontically treated teeth (3.0% and 2.7%) than the study population average (2.3%). In addition, the frequency of endodontically treated teeth with AP in the 40-49 year age group was also significantly different (P < 0.001) than in the study population average.

**Table 3:** Distribution of total number of teeth, teeth with AP, ET and AP/ET according to the age group

Age group	No of teeth	Teeth with AP (%)	Teeth with ET (%)*	Teeth w. AP/ET (%)**
< 20	408	30 (7.4%)	5 (1.2%)	2 (40.0%)
20-29	1007	98 (9.7%)	30 (3.0%)	15 (50.0%)
30-39	1584	181 (11.4%)	37 (2.3%)	16 (43.2%)
40-49	594	94 (15.8%)	16 (2.7%)	9 (56.3%)
50-59	340	66 (19.4%)	2 (0.6%)	-
≥ 60	198	40 (20.2%)	5 (2.5%)	2 (40.0%)

**Total**	**4131**	**509 (12.3%)**	**95 (2.3%)**	**44 (46.3%)**

*P < 0.01, **P < 0.001.

AP, apical periodontitis; ET, endodontically treated teeth; AP/ET, apical periodontitis in endodontically treated teeth

The prevalence of AP in the anterior region was 12.4%, compared with 12.2% in posterior teeth (Table 4). More endodontically treated teeth were found in the posterior region than in the anterior region and this difference was statistically significant (p < 0.001). However, the difference between the prevalence of AP in anterior and posterior regions was not significant (P > 0.05).

**Table 4:** Distribution of total number of teeth, teeth with AP, ET and AP/ET regarding the region

Region	No of teeth	Teeth with AP (%)	Teeth with ET (%)	Teeth w. AP/ET (%)
Anterior	2050	255 (12.4%)	28 (1.4%)	10 (35.7%)
Posterior	2081	254 (12.2%)	67 (3.2%)	34 (50.7%)

**Total**	**4131**	**509 (12.3%)**	**95 (2.3%)**	**44 (46.3%)**

AP, apical periodontitis; ET, endodontically treated teeth; AP/ET, apical periodontitis in endodontically treated teeth

The prevalence of AP was not significantly different between maxillary teeth and mandibular teeth (Table 5). There were significantly more endodontically treated teeth in the maxilla than in the mandible (P < 0.001). The difference in the prevalence of AP in endodontically treated maxillary and mandibular teeth was not statistically significant.

**Table 5:** Distribution of total number of teeth, teeth with AP, ET and AP/ET regarding the jaw

Jaw	No of teeth	Teeth with AP (%)	Teeth with ET (%)*	Teeth w. AP/ET (%)
Maxilla	2100	290 (13.8%)	68 (3.2%)	31 (45.6%)
Mandible	2031	219 (10.8%)	27 (1.3%)	13 (48.1%)

**Total**	**4131**	**509 (12.3%)**	**95 (2.3%)**	**44 (46.3%)**

*P < 0.001

AP, apical periodontitis; ET, endodontically treated teeth; AP/ET, apical periodontitis in endodontically treated teeth

The relationship between the quality of root filling and periapical status is presented in Table 6. The percentage of adequately-filled teeth with AP was 20.7%, whereas the percentage of teeth with inadequate root filling and AP was 57.6% (P = 0.002, Odds ratio: 5.20; CI:1.87-14.46).

**Table 6:** Quality of root filled teeth and the relation to the periapical status, percentage for healthy/diseased

Root filled teeth	Healthy (%)	Diseased (%)	Total (%)	P
Adequate	23 (79.3%)	6 (20.7%)	29 (30.5%)	
Inadequate	28 (42.4%)	38 (57.6%)	66 (69.5%)	0.002

**Total**	**51 (53.7%)**	**44 (46.3%)**	**95 (100.0%)**	

Chi-square test (P = 0.002, Odds ratio:5.20; CI:1.87-14.46.)

## Discussion

The findings of this study demonstrate that AP is more prevalent in the surveyed Kosovar population (12.3%) than in other populations [9,10,19,20,25]. This result could arise for a number of reasons. First, the oral status of Kosovar adults is unsatisfactory, due to insufficient oral hygiene leading to a large numbers of carious teeth, and/or inadequate quality of fixed dentures. Second, socioeconomic factors may have a role in oral hygiene and, third, the absence of programs for integrated prevention and control of dental caries could exacerbate these problems and lead to more advanced dental and periodontal problems.

In this study, the total percentage of endodontically treated teeth was 2.3%, which is low compared with the results of some other studies [10,11,26,29], in which the range was between 6.8 and 18.5%. This could be a consequence of the survey population being unrepresentative of the whole country and/or to differences in the socio-economic factors and the provision of dental care services in these various other countries. In contrast, our data are in agreement with a number of previous studies that found the prevalence of endodontically treated teeth to range between 1.3 and 4.8% [9,13,19,21,22,25,30].

The results of our study showed that the prevalence of AP in endodontically treated teeth is 46.3%. This prevalence was lower than that reported in Spain (64.5%) [25] but remain considerably higher than that reported in Portugal (22%) [30], Ireland (25%) [24], the United States (31.3%) [20], France (31.5%) [28]. The high rate of AP in endodontically teeth may be the result of inadequate endodontic treatment provided by general dentists in Kosovo (and Spain).

We have also shown an age-related increase in the frequency of AP-affected teeth (Table 3), with prevalence being highest in the older groups. Other studies have also demonstrated that the prevalence of AP increases with age [5,20,25,31,32]. Younger people tend to visit the dentist more often compared with the elderly, and thus have a lower incidence of caries and periodontal diseases. There was no significant association of gender with the frequency of either AP alone or AP in endodontically treated teeth, in agreement with the findings of previous studies [5,25,26,33-36].

When sub-divided by whether the teeth are mandibular or maxillary, our data regarding the frequency of AP and endodontically treated teeth are comparable with those of other studies [37]. These studies found no difference between the maxilla and the mandible in the average percentage of teeth with AP, yet the percentage of endodontically treated maxillary teeth was twice that of endodontically treated mandibular teeth. However, the incidence of AP in maxillary teeth was found to be almost twice that in the mandible [11,20,25,26,36,38].

Regarding the anterior/posterior region, there was no significant difference in the prevalence of AP, but endodontic treatment of posterior teeth was associated with significantly more AP than the same treatments in anterior teeth (Table 4), in agreement with previous studies [25,36].

The prevalence of AP was influenced by the quality of the root filling. The measurement and categorization of the quality of the root filling differs among studies. Some studies use only length for determining technical quality [10,19,39]. Other studies have used both length and adequate seal, either reporting both parameters individually [13] or combining the length and seal into a single amalgamated value [12]. Petersson et al. reported only on complete/incomplete obturation and whether there was any evidence of overfilling [40]. In our study, the criteria used for the evaluation of the quality of root filling were modified slightly from those described by Tronstad et al. [27] and Tavares et al. [28].

The quality of the root filling was frequently unsatisfactory. AP was present in 46.3% of endodontically treated teeth. Only 30.5% of the root filled teeth fulfilled the criteria for an acceptable root canal filling, which is low compared with other studies [10,12,19,20,22,30]. Although the methods/parameters used to evaluate the quality of root canal filling were not the same as other studies, we are confident that the poor quality of root canal filling observed in our study are the result of low standards and/or poor technique in root canal procedures. This situation arises because, in Kosovo, endodontic treatment is performed by general dentists; endodontic specialists with additional training and expertise could raise the general standard of root canal treatments but there are currently very few practicing dentists with these skills in Kosovo.

## Conclusions

Continuing medical education efforts to train future and current dental practitioners, extended support for programs in caries control and prevention, and general improvement in the socio-economic status of the population would make a significant contribution to reducing the prevalence of AP associated with endodontic treatment in the population of Kosovo. Further studies of dental health involving larger samples that cover more regions of Kosovo will help to identify public dental health problems, an essential step in improving the general health status of the citizens of this country.

## Competing interests

The authors declare that they have no competing interests.

## Authors' contributions

BK was the head of the study and made substantial contributions to conception and design of the study. MS and AK were radiographic examiners. ED performed the statistical analysis. BK, VH, MS, AK, ED and LK participated in manuscript design and writing. All authors read and approved the final manuscript.

## Pre-publication history

The pre-publication history for this paper can be accessed here:

http://www.biomedcentral.com/1472-6831/11/32/prepub
